# Physiological properties of beetroot crisps applied in standard and dyslipidaemic diets of rats

**DOI:** 10.1186/1476-511X-10-178

**Published:** 2011-10-14

**Authors:** Monika Wroblewska, Jerzy Juskiewicz, Wieslaw Wiczkowski

**Affiliations:** 1 Institute of Animal Reproduction and Food Research of the Polish Academy of Sciences in Olsztyn, Poland

**Keywords:** Beetroot, rats, dyslipidaemic diet, gastrointestinal tract, atherogenic index

## Abstract

**Background:**

The objective of the present study was to examine the influence of adding various amounts beetroot (*Beta vulgaris*) crisps on gastrointestinal function, antioxidant status and blood and liver lipid profiles in a high fat diet-induced dyslipidaemic rat model;

**Results:**

The intake of a dyslipidaemic diet increased the serum total cholesterol, total cholesterol-to-HDL-cholesterol ratio, atherogenic index, hepatic total cholesterol and triacylglycerols, suppressed production of short-chain fatty acids and decreased total antioxidant status and blood glutathione peroxidase activity. Oral administration of all tested amounts of beetroot crisps prevented the rise in serum total cholesterol and triacylglycerols levels. The treatment with the addition of 3% crisps also decreased hepatic total cholesterol level and activity of AST in serum. The experimental addition of crisps likewise resulted in a tendency towards a higher total SCFA pool and activity of glutathione peroxidase and a lower serum glucose level (*p *= 0.080, *p *= 0.061 and *p *= 0.067, respectively);

**Conclusions:**

Results of the presented study suggest that the addition of beetroot crisps could alleviate metabolic changes in dyslipidaemic diet-administered rats.

## Background

A Western diet heavy in meat, fried foods and refined grains increases the risk of developing metabolic syndrome, heart problems, stroke and type 2 diabetes. The link between diet and chronic disease is well documented. Heart disease, the main cause of death in Western countries, is greatly influenced by diet, especially by the amount and type of fat consumed. Nutritionists recommend diets rich in fruits and vegetables for good health and for reducing the risk of heart disease and some types of cancers. The beneficial effects of fruits, vegetables and a Mediterranean diet are likely due to many components, including fibre and micronutrients [1]. The fundamental active agents for reducing the risk of chronic diseases are antioxidants [2]. Over the last several years, increasing attention has been paid to the antioxidant activity of plant pigments such as caretonoids, anthocyanins and, recently, the betalains [3,4]. The most heavily studied betalain is betanin aglycone, obtained from the extract of beet juice. Beetroot is mainly consumed as a pickled or canned preserve, a cooked vegetable or sometimes a juice. In Poland, red beets are popular vegetables with a per capita consumption of approximately 6 kg/year. Results from several *in vitro *studies have demonstrated that betalains from beetroots possess powerful antiradical and antioxidant activity [5-8]. In addition, a significant tumour inhibitory effect has been shown to accompany a dietary treatment with beetroot ingestion [9]. Lammers [10] hypothesized that betanin is non-perturbing to cellular metabolism, highly compatible with enzyme function and steadies cellular metabolic function under various kinds of stress in animal tissue. Introduction of novel products, mainly lacto-fermented juice and crisps, has presumably contributed to the recent growth in the intake of beetroot. Some epidemiological studies have shown a contrary correlation between fruit/vegetable intake and the risk of cardiovascular disease, especially when the blood lipid profile is considered [11,12].

In this study, the hypothesis that the consumption of a diet containing beetroot crisps could beneficially affect gastrointestinal function, antioxidant status of the body, and blood and liver lipid profiles was verified. To test this hypothesis, different amounts of beetroot crisps were applied to a Western-type and a dyslipidaemic diet.

## Materials and methods

Beetroot crisps were obtained from Paula Co. (Kalisz, Poland) and contained 14.6 g of crude protein, 48.4 g of carbohydrates and 0.4 g fat per 100 g of crisps.

The extraction, quantification and identification of betalains from beetroot crisps is described below. About 0.10 g of pulverized red beet crisps was extracted with 1 ml of distilled water by with triplicate 60-s sonication and 60-s vortexing. After centrifugation (13200 × *g *at 4°C, 5 min), the supernatant was collected in a 5-ml flask. These steps were repeated 5 times. Quantification of betalains was determined by a spectrophotometric method according to the modified method of Stintzing [13]. Briefly, before analysis aliquots of red beet crisps extract was centrifuged (8400 × *g*, 5 min). Next, the sample was dissolved in McIlvaine buffer (pH 6.5) to obtain an absorbance (A) of 0.8 ≤ A ≤ 1.0. The betacyanins and betaxanthins contents were calculated as betanin-equivalents and vulgaxanthin-equivalents, respectively, using the following formula: [mg/g dry matter] = (A*R*MW*V*1000)/ε*W, where "A" is the absorption at 538 nm and 480 nm for betacyanins and betaxanthins, respectively, "R" is the dilution factor, "V" is the volume of extract (L), "W" is the sample weight (g), MW is the molecular weight (550 g/mol for betanin, 339 g/mol for vulgaxanthin) and "ε" is the molar extinction coefficients (60000 L/mol*cm at λ = 538 nm for betanin, 48000 L/mol*cm at λ = 480 nm for vulgaxanthin). Chromatographic determination of betalains were done on a Shimadzu HPLC system (Kyoto, Japan) consisting of two pumps (LC-10AD_VP_), a DAD detector (SPD-M10 A_VP_) set at 538 nm and 480 nm, a MS detector (QP8000α), an autosampler (SIL-10 AD_VP_) set to 10 μL injection, a column oven (CTO-10 AS_VP_) and a system controller (SCL-10 A_VP_). Before HPLC injection, the extracts were centrifuged (20 min, 13200 × *g*). All chromatographic determinations were performed at 25°C with the flow rate of 0.2 mL/min on C-18 BDS Hypersile-Keystone 3 μ 100 × 4 mm column (Thermo, Waltham USA). The elution was done using a solvent gradient system consisting of solvent A (0.1% formic acid aqueous solution) and solvent B (0.1% formic acid acetonitrile solution). Gradients were as follows: 2-17-80-80-2-2% B at gradient times, t_G _= 0-35-36-42-43-60 min. Betalains were identified based on the UV-visible spectrum, the presence of the respective ion (m/z values) and previously published data [13-15]. The mass spectrometer with electrospray ionization (ESI) worked in the positive mode with the following parameters: CDL temperature of 260°C, CDL voltage of 70V, probe voltage of 4.5 kV, nebulizer gas (N_2_) flow of 4.5 L/min, and defragmentation voltage of 50 V. All determinations were performed in triplicate. The use of animals was conducted in compliance with European guidelines for the care and use of laboratory animals and was approved by the Ethical Committee for Animal Experiments in the northeastern region of Poland. The experiment was performed on 64 male Wistar rats aged approximately 4 weeks (body weight 103.8 ± 3.92 g). The experimental diets were administered for 4 weeks to 8 rats per group housed individually in Plexiglass cages. The prevailing environmental temperature was 21-22°C, relative humidity of 50-70% with a 12 h light-dark cycle and intensive ventilation of rooms (15×/h).

The animals were allowed access to experimental diets and water *ad libitum*. The composition of the experimental diets is shown in Table 1. All diets contained 14.8% casein, 5% cellulose, 0.2% DL-methionine and 0.2% vitamin-mineral mixture AIN-93G [16]. The standard basal diet containing 8% soybean oil and 0.3% cholesterol was supplemented with different amounts of beetroot crisps: 0, 0.3, 1 or 3%. The dyslipidaemic basal diet was prepared with the aid of lard instead of oil, an extended amount of cholesterol - 1%, and 0.5% cholic acid, and was supplemented with the same amounts of crisps.

**Table 1:** Composition of experimental diets, %

	**B**_**0**_		**B**_**0.3**_		**B**_**1**_		**B**_**3**_	
	
	S	D	S	D	S	D	S	D
Casein	14.8	14.8	14.8	14.8	14.8	14.8	14.8	14.8
DL-methionine	0.2	0.2	0.2	0.2	0.2	0.2	0.2	0.2
Cellulose	5	5	5	5	5	5	5	5
Beetroot crisps	-	-	0.3	0.3	1	1	3	3
Soybean oil	8	-	8	-	8	-	8	-
Lard	-	8	-	8	-	8	-	8
Cholic acid	-	0.5	-	0.5	-	0.5	-	0.5
Cholesterol	0.3	1	0.3	1	0.3	1	0.3	1
Mineral mix^1^	3	3	3	3	3	3	3	3
Vitamin mix^1^	2	2	2	2	2	2	2	2
Maize starch	66.7	65.5	66.4	65.2	65.7	64.5	63.7	62.5

^1^According to Reeves (1997)

At the end of the feeding experiment, the experimental animals were anaesthetized with sodium pentobarbitone, blood was taken from the caval tail vein, and serum was then obtained after clotting. The gastrointestinal tract was excised immediately and weighed. The pH of in the contents of the stomach, small intestine, caecum and colon was measured using a microelectrode and a pH/ION meter (model 301, Hanna Instruments). Samples of fresh digestive tract contents were used for immediate analysis (dry matter content and ammonia); the rest was transferred to tubes and stored at -40°C. Dry matter of the digesta was determined at 105°C. Ammonia extracted and trapped in a solution of boric acid in Conway dishes was determined by direct titration with sulphuric acid [17]. Glycolytic activity in the caecal digesta was measured by the rate of release of *p*- or *o*-nitrophenol from their nitrophenylglucosides according to the modified method of Djouzi and Andrieux [18] described by Juśkiewicz and co-workers [19]. The following substrates were used: *p*-nitrophenyl-*β*-D-glucuronide for *β*-glucuronidase and *p*-nitrophenyl-*β*-D-glucopyranoside for *β*-glucosidase. The reaction mixture contained 0.3 ml of substrate solution (5 mM) and 0.2 ml of a 1:10 (v/v) dilution of the caecal sample in 100 mM phosphate buffer (pH 7.0) after centrifugation at 10,000 × *g *for 15 min. Incubations were carried out at 37°C for 10 min, and *p*-nitrophenol was quantified at 400 nm after the addition of 2.5 ml of 0.25 M cold sodium carbonate. Enzymatic activity (*β*-glucosidase and *β*-glucuronidase) was expressed as μmol product formed per min (IU) per g digesta in the caecal sample. The concentration of short-chain fatty acids (SCFA) was analysed by gas chromatography: Shimadzu GC-14A with a glass column 2.5 m × 2.6 mm, containing 10% SP-1200/1% H_3_PO_4 _on 80/100 Chromosorb W AW with column temperature 110°C, detector FID temperature 180°C, injector temperature 195°C. The caecal digesta were weighed (sample of about 0.2 g), mixed with 0.2 ml of formic acid, diluted with deionised water and centrifuged at 10,000 × *g *for 5 min [20]. The supernatant was decanted for injection into the gas chromatograph. Diagnostic kits (Alpha Diagnostics, Poland) were used for determination of the concentration of glucose, cholesterol (total and HDL), triglycerides and activity of alanine aminotransferase (ALT) and aspartate aminotransferase (AST) in the serum. HDL cholesterol was measured after selective precipitation of low and very low-density serum lipoproteins with polypropylene glycol (PEG-600) and further removal by centrifugation. The atherogenic index was calculated using the formula: atherogenic index = TC-HDL/HDL described by Aziz [21]. Superoxide dismutase (EC 1.1.5.1; SOD) and glutathione peroxidase (EC 1.11.1.9; GPx) activities in erythrocyte lysates and total antioxidant status (TAS) were assayed using kits from Randox Laboratories Ltd. (Crumlin, United Kingdom). The principle of the TAS assay is the incubation of ABTS with peroxidase (metmyoglobin), which results in the production of a radical cation. This species is a blue-green colour and can be detected at 600 nm. In the added sample, the antioxidants suppress the production of this colour proportionally to their concentration.

Total hepatic cholesterol and triglycerides were measured in kidneys and liver using standard diagnostic kits (Alpha Diagnostics, Poland) after lipid extraction by the Folch method.

### Statistical analysis

All data were analyzed using two-way ANOVA with the aid of Statistica 6.0 for Windows (StatSoft Corp, Krakow, Poland). Data are expressed as means. Multiple comparisons of the means were performed using the Duncan Multiple Range Test at a 5% probability.

## Results

Betalains analysis in red beet crisps showed that betacyanins content was 4.10 ± 0.05 mg/g of the dry matter, whereas betaxanthin content was 2.80 ± 0.07 mg/g of the dry matter. Betacyanins were present as betanin (57%), isobetanin (35%) and neobetanin (1.4%). In the case of betaxanthins, the predominant compound was vulgaxanthin (70%).

After the experimental period (4 weeks), final body weight (BW) of the rats fed standard diets were higher as compared to those fed dyslipidaemic diets (267.1 vs. 248.8 g, *p *< 0.05), whereas the experimental dietary application of the crisps did not affect the final BW of the animals (Table 2). The type of diet significantly affected the pH value in the stomach, ileum and caecum (dyslipidaemic > standard, *p *< 0.05). The highest dietary addition of beetroot crisps (3% of a diet) caused a significant alkalization of ileal digesta as compared to other treatments. Rats fed dyslipidaemic diets were characterized by decreased bulk and dry matter concentrations in the caecal contents as well as elevated concentrations of caecal ammonia (*p *< 0.05 vs. standard diet). When the application of beetroot crisps is considered, the highest and the lowest caecal dry matter concentrations were observed in the B_0 _and B_3 _treatments (*p *< 0.05 vs. B_1_, B_3 _and *p *< 0.05 vs. B_0_, B_0.3_, respectively). The activity of bacterial β-glucosidase and β-glucuronidase was significantly diminished in the caecum of dyslipidaemic rats.

**Table 2:** Final body weights and parameters of gastrointestinal tract of rats fed experimental diets

	Body weight, g	*pH*			*Caecal indices*				*Activity of bacterial enzymes in caecal digesta*	
	
		stomach	ileal	caecal	**tissue, g/100 g b.w**.	**digesta, g/100 g b.w**.	ammonia, mg/100 g	dry matter, %	β-Glucosidase	β-Glucuronidase
*Subgroup*										
B_0_S	265.6	3.67	6.48	6.74	0.227	0.965	3.27	24.88	0.433	0.94
B_0.3_S	262.8	3.68	6.55	6.71	0.239	1.099	3.15	23.54	0.490	0.87
B_1_S	264.8	3.61	6.48	6.92	0.231	1.073	3.55	23.17	0.380	1.10
B_3_S	275.3	3.54	6.85	6.85	0.239	1.085	3.07	22.00	0.393	0.87
B_0_D	248.1	3.89	6.70	7.11	0.238	0.706	4.35	22.25	0.059	0.36
B_0.3_D	245.7	3.74	6.92	7.11	0.231	0.717	4.12	22.41	0.048	0.35
B_1_D	250.8	4.28	6.68	7.13	0.263	0.807	4.21	20.20	0.039	0.39
B_3_D	250.6	4.10	6.99	7.17	0.243	0.937	3.77	18.78	0.035	0.36

SEM	2.081	0.072	0.036	0.030	0.006	0.031	0.093	0.321	0.029	0.044

*Chips (C)*										
B_0_	256.9	3.78	6.59^b^	6.93	0.233	0.835	3.81	23.56^a^	0.246	0.65
B_0.3_	254.3	3.71	6.74^b^	6.91	0.235	0.908	3.64	22.98^ab^	0.269	0.61
B_1_	257.8	3.94	6.58^b^	7.03	0.247	0.940	3.88	21.69^bc^	0.210	0.74
B_3_	262.9	3.82	6.92^a^	7.01	0.241	1.011	3.42	20.39^c^	0.214	0.62
P value (C)	0.374	0.681	< 0.05	0.188	0.830	0.126	0.159	< 0.05	0.525	0.214

*Diet (D)*										
Standard	267.1^a^	3.62^b^	6.59^b^	6.81^b^	0.234	1.055^a^	3.26^b^	23.40^a^	0.424^a^	0.94^a^
Dyslipidemic	248.8^b^	4.00^a^	6.82^a^	7.13^a^	0.244	0.792^b^	4.11^a^	20.91^b^	0.045^b^	0.36^b^
P value (D)	< 0.05	< 0.05	< 0.05	< 0.05	0.402	< 0.05	< 0.05	< 0.05	< 0.05	< 0.05

P value (CxD)	0.751	0.372	0.564	0.475	0.667	0.470	0.720	0.433	0.707	0.454

^abc ^- values within each row with the same superscript are not different at P ≤ 0.05

S-standard groups; D-dyslipidemic groups, B_0 _-groups without beetroot crisps, B_0.3_, B_1_, B_3 _-groups with 0.3, 1 and 3% beetroot crisps respectively

In the present investigation, feeding dyslipidaemic diets significantly lowered total SCFA as well as acetic, propionic, butyric and valeric acid concentrations (Table 3). The supplementation of 3% beetroot crisps was accompanied by a significant decrease in caecal isovaleric acid concentration in comparison to other treatments (*p *≤ 0.05). The dietary supplementation with 1 and 3% of beetroot crisps caused a higher acetate proportion compared to the group without crisps added. The type of diet significantly influenced the SCFA profile; dyslipidaemic groups were characterized by a lower percentage of butyric acid and a higher ratio of propionic and acetic acid in comparison to the rats fed standard diets. In addition, two-way ANOVA revealed that among the dyslipidaemic groups the highest dietary addition of beetroot crisps resulted in a diminished ratio of butyrate in the SCFA profile as compared to the B_0_D group (see significant interaction C×D). The experimental addition of beetroot crisps in amounts of 0.3-3% of a diet resulted in a significant increase in the caecal acetate pool (expressed as μmol/100 g of BW) as well as a tendency towards a higher total SCFA pool (*p *= 0.080). Moreover, total and individual SCFA pools were about twofold higher in the standard diet treatments than in the dyslipidaemic groups (*p *≤ 0.05).

**Table 3:** Short chain fatty acids concentration (SCFA), pool and profile in the caecal digesta of rats fed experimental diets

	*Concentration, μmol/g*							*Profile, μmol/100 μmol SCFA*			*SCFA pool, μmol/100 g bw*						
	
	C2	C3	C4i	C4	C5i	C5	Total	C2	C3	C4	C2	C3	C4i	C4	C5i	C5	Total
*Subgroup*																	
B_0_S	67.4	18.6	0.85	12.7	1.37	1.58	102.5	66	18	13^a^	64.4	17.9	0.83	12.3	1.33	1.52	98.3
B_0.3_S	76.3	19.5	0.85	13.8	1.32	1.69	113.4	67	17	12^a^	83.6	21.6	0.93	15.2	1.46	1.85	124.7
B_1_S	77.3	18.3	0.82	13.3	1.28	1.62	112.7	68	16	12^a^	82.8	19.9	0.91	13.9	1.37	1.71	120.6
B_3_S	75.2	17.8	0.65	14.7	0.88	1.58	110.8	68	16	13^a^	80.7	19.3	0.71	15.8	0.99	1.70	119.3
B_0_D	53.5	15.08	0.93	6.70	1.27	0.78	78.22	68	19	9^b^	39.3	11.3	0.67	5.0	0.92	0.55	57.7
B_0.3_D	59.6	16.29	0.89	6.95	1.33	1.03	86.14	69	19	8^bc^	42.2	11.5	0.62	4.8	0.89	0.71	60.70
B_1_D	56.5	15.10	0.73	6.60	1.09	0.97	81.01	70	19	8^bc^	46.2	12.2	0.61	5.3	0.88	0.79	66.0
B_3_D	56.05	13.94	0.65	5.60	0.88	0.81	77.92	72	18	7^c^	50.7	13.0	0.59	5.0	0.81	0.78	70.9

SEM	1.695	0.391	0.037	0.501	0.050	0.057	2.474	0.364	0.262	0.330	2.798	0.734	0.042	0.651	0.053	0.074	4.192

*Chips (C)*																	
B_0_	60.4	16.9	0.89	9.7	1.32^a^	1.18	90.37	67^b^	19	11	51.9^b^	14.6	0.75	8.6	1.12	1.04	78.0
B_0.3_	68.0	17.9	0.87	10.3	1.33^a^	1.36	99.78	68^ab^	18	10	62.9^a^	16.6	0.78	10.0	1.17	1.28	92.7
B_1_	66.9	16.7	0.77	10.0	1.19^a^	1.29	96.84	69^a^	17	10	64.5^a^	16.0	0.76	9.6	1.13	1.25	93.3
B_3_	65.6	15.9	0.65	10.2	0.88^b^	1.19	94.38	70^a^	17	10	65.7^a^	16.2	0.65	10.4	0.90	1.24	95.1
P value (C)	0.180	0.197	0.093	0.735	< 0.05	0.222	0.256	< 0.05	0.076	0.738	< 0.05	0.633	0.691	0.131	0.164	0.126	0.080

*Diet (D)*																	
Standard	74.0^a^	18.6^a^	0.79	13.6^a^	1.21	1.62^a^	109.9^a^	67^b^	17^b^	12^a^	77.9^a^	19.7^a^	0.85^a^	14.3^a^	1.29^a^	1.69^a^	115.7^a^
Dyslipidemic	56.4^b^	15.1^b^	0.80	6.5^b^	1.14	0.90^b^	80.8^b^	70^a^	19^a^	8^b^	44.6^b^	12.0^b^	0.62^b^	5.0^b^	0.87^b^	0.71^b^	63.8^b^
P value (D)	< 0.05	< 0.05	0.906	< 0.05	0.457	< 0.05	< 0.05	< 0.05	< 0.05	< 0.05	< 0.05	< 0.05	< 0.05	< 0.05	< 0.05	< 0.05	<0.05

P value (CxD)	0.794	0.982	0.874	0.062	0.852	0.818	0.792	0.458	0.805	< 0.05	0.385	0.628	0.810	0.102	0.478	0.727	0.433

^abc ^- values within each row with the same superscript are not different at P ≤ 0.05

S-standard groups; D-dyslipidemic groups, B_0 _-groups without beetroot crisps, B_0.3_, B_1_, B_3 _-groups with 0.3, 1 and 3% beetroot crisps respectively

The content of beetroot crisps substantially decreased the serum triacylglycerols and total cholesterol level in rats (*p *≤ 0.05, Table 4). In the latter case, it was due to hypocholesterolemic action of dietary crisps in the dyslipidaemic groups (see significant interaction C×D, *p *= 0.05). The highest and the lowest dietary addition of beetroot crisps also resulted in a tendency towards decreased serum glucose concentration and atherogenic index value (*p *= 0.067 and *p *= 0.095, respectively). The B_3 _treatment was associated with a decreased serum AST activity in comparison to treatment B_1_. The treatments with the addition of crisps tended to increase blood GPx activity (*p *= 0.061). The rats fed dyslipidaemic diets were characterized by decreased concentrations of serum triacylglycerols and HDL-cholesterol, diminished TAS of the serum and blood GPx activity as well as increased serum level of total cholesterol, total cholesterol-to-HDL cholesterol ratio and atherogenic index level. Dyslipidaemic rats were also characterized by increased activities of serum AST and ALT (*p *≤ 0.05). The significant interaction revealed that among the dyslipidaemic rats those fed 3 and 0.3% crisps-containing diets were characterized by decreased ALT activity (*p *< 0.05 vs. B_0_D, B_1_D and *p *< 0.05 vs. B_0_D group, respectively).

**Table 4:** Activity and concentration a biochemical indices of serum and blood of rats fed experimental diets

	*Concentration*							Activity of enzymes			
	
	Glucose, mmol/L	Triacylglyceroles, mmol/L	Total Cholesterol, mmol/L	HDL Cholesterol, mmol/L	Total Cholesterol/HDL Cholesterol	Atherogenic index	Total Antioxidant Status, mmol/L	ALT, U/L	AST, U/L	GPx, U/mL	SOD, U/mL
*Subgroup*											
B_0_S	15.12	2.64	2.77^c^	1.31	2.20	1.20	0.952	28^d^	110	31388	276
B_0.3_S	14.61	1.95	2.46^c^	1.30	1.93	0.93	0.921	37^d^	110	33193	281
B_1_S	14.13	2.10	2.62^c^	1.28	2.08	1.08	0.924	37^d^	111	34946	289
B_3_S	13.37	2.19	2.64^c^	1.32	2.04	1.04	0.954	37^d^	109	33309	287
B_0_D	15.39	0.91	12.70^a^	0.84	15.41	14.41	0.816	62^a^	161	27605	278
B_0.3_D	13.49	0.91	10.24^b^	1.01	10.99	9.99	0.836	55^bc^	162	30662	272
B_1_D	14.11	0.80	10.27^b^	0.78	13.29	12.29	0.900	57^ab^	171	30707	272
B_3_D	13.08	1.02	10.22^b^	0.86	12.69	11.69	0.871	49^c^	142	30078	270

SEM	0.263	0.096	0.555	0.038	0.779	0.779	0.009	1.614	3.718	489.44	3.528

*Chips (C)*											
B_0_	15.26	1.77^a^	7.74^a^	1.07	8.80	7.80	0.884	45	136^ab^	29496	277
B_0.3_	14.05	1.43^b^	6.35^b^	1.15	6.46	5.46	0.879	46	136^ab^	31927	277
B_1_	14.12	1.45^b^	6.44^b^	1.03	7.69	6.69	0.912	47	141^a^	32826	281
B_3_	13.22	1.61^b^	6.43^b^	1.09	7.37	6.37	0.912	43	125^b^	31693	279
P value (C)	0.067	< 0.05	< 0.05	0.402	0.105	0.105	0.198	0.283	< 0.05	0.061	0.979

*Diet (D)*											
Standard	14.31	2.22^a^	2.62^b^	1.30^a^	2.06^b^	1.06^b^	0.938^a^	35^b^	110^b^	33209^a^	283
Dyslipidemic	14.02	0.91^b^	10.86^a^	0.87^b^	13.10^a^	12.10^a^	0.856^b^	56^a^	159^a^	29763^b^	273
P value (D)	0.579	< 0.05	< 0.05	< 0.05	< 0.05	< 0.05	< 0.05	< 0.05	< 0.05	< 0.05	0.160

P value (CxD)	0.808	0.077	0.050	0.435	0.179	0.179	0.067	< 0.05	0.092	0.906	0.785

^abc ^- values within each row with the same superscript are not different at P ≤ 0.05

Atherogenic index was calculated as fallow TC-HDL/HDL

S-standard groups; D-dyslipidemic groups, B_0 _-groups without beetroot crisps, B_0.3_, B_1_, B_3 _-groups with 0.3, 1 and 3% beetroot crisps respectively

Dyslipidaemic diets enhanced hepatic triacylglycerols and total cholesterol levels (Table 5). Diet supplementation with 3% beetroot crisp decreased liver cholesterol levels in comparison to other treatments, but only when dyslipidaemic groups are considered.

**Table 5:** Concentration of hepatic cholesterol and triacylglyceroles of rats fed experimental diets

	*Liver*	
	
	Triacylglyceroles, mg/g	Total Cholesterol, mg/g
*Subgroup*		
B_0_S	10.63	7.43^c^
B_0.3_S	11.76	8.81^c^
B_1_S	9.53	7.29^c^
B_3_S	9.23	6.99^c^
B_0_D	16.69	29.49^a^
B_0.3_D	15.23	30.43^a^
B_1_D	13.23	29.62^a^
B_3_D	13.14	23.65^b^

SEM	0.617	1.364

*Chips (C)*		
B_0_	13.66	18.46^a^
B_0.3_	13.49	19.62^a^
B_1_	11.38	18.45^a^
B_3_	11.19	15.32^b^
P value (C)	0.256	< 0.05

*Diet (D)*		
Standard	10.29^b^	7.63^b^
Dyslipidemic	14.57^a^	28.30^a^
P value (D)	< 0.05	< 0.05

P value (CxD)	0.843	< 0.05

^abc ^- values within each row with the same superscript are not different at P ≤ 0.05

## Discussion

Consumption of beetroot and its products has many possible health benefits, linked to a high content of betalains. In our study, the content of beetroot crisps in the rat diet was 0.3, 1 or 3%, which corresponds to a daily intake of 18, 60 and 180 g beetroot crisps for adult consumers with an average body weight of 70 kg.

In the current study, addition of beetroot crisps did not significantly affect final body weight, but final body weights were significantly influenced by fat type supplementation. In a study by Danicke [22], feeding male broiler chickens a diet containing tallow decreased the final body weights of the birds. Animals fed a high beef fat diet ate less food and developed more and larger intestinal tumours with more metastases to the abdominal cavity, lungs and liver [23]. On the other hand, in experiment by Moundras [24], a diet modified by the addition of 0.3% cholesterol did not significantly change the final body weight of rats. In this study, a significant decrease in pH of digesta in the ileum and dry matter content in the caecum digesta, a slight increase of caecum digesta mass (i.e., by about 8-21%) when compared to the control and comparable ammonium concentrations showed that diets with beetroot crisps slightly affected indices of caecal function. In this experiment, dylipidaemic diet treatment lowered caecal digesta weight and enhanced the pH value in the gastrointestinal tract. The results of our investigation are different from those of Moundras [24], who did not observe significant changes in caecal pH. Addition of red beet crisps to the diet had no influence on the activity of bacterial enzymes in the caecum digesta, although it is known that endogenous β-glucosidase participates in biotransformation of polyphenolics in the alimentary tract [25], and higher polyphenolic content may evoke its lower activity in intestinal digesta [26]. A lower activity of microbial β-glucuronidase in the digesta is desirable as this enzyme participates in the activation of many procarcinogenic compounds [27]. The dyslipidaemic diet significantly affected the activity of β-glucosidase and β-glucuronidase, and these effects were accompanied by a reduction of SCFA production, higher alkalization of the digesta and lower bulk and dry matter concentrations in the caecal contents, which may indicate inhibition of growth of bacteria in the gastrointestinal tract.

Our results showed that the supplementation of diets with beetroot crisps marginally enhanced the total SCFA pool compared to the control group pool but did not affect the caecal pH. This study identified unwanted changes of SCFA concentration under the influence of lard and cholesterol addition to the diet. The dyslipidaemic diet decreased the ratio of butyric acid in the SCFA profile and increased the acetic and propionic ratios. The addition of lard and cholesterol to the diet decreased the SCFA pool compared to the control groups, but this effect was not observed by Moundras [24]. It is well known that SCFAs are the main product of bacterial fermentation in the large intestine of animals and humans. All SCFAs are rapidly absorbed from the hindgut and stimulate water and salt absorption. SCFAs play an important role in the function of the large bowel as an energy source for the colonic epithelium. Butyrate, which regulates epithelial cell growth, is especially important for health of the large bowel [28].

In the reported study, diet supplementation with 0.3 and 1% crisps decreased the triacylglycerol level by about 20%. Glucose level was similarly affected by tea catechins in the experiment by Kao and co-authors [29] conducted with Sprague-Dawley rats and 5-month-old mice with 1% tea catechin [30]. In this work, the application of a dyslipidaemic diet led to the deterioration of the biochemical indices of serum in rats, reflected in an enhanced total cholesterol level (4 fold) and higher activity of transaminases and at the same time lower HDL cholesterol level. Moundras [24] observed an increase in total cholesterol level in a group of rats fed a diet with cholesterol and significantly higher serum triglyceride concentrations. Mandukhail also showed that a dyslipidaemic diet increased serum total cholesterol, total cholesterol-to-HDL-cholesterol ratio and atherogenic index [31]. High fat treatment resulted in oxidative stress in rats thus enhanced oxidation of low-density lipoprotein, which plays a key role in the genesis of atherosclerosis. Antioxidants such as betalains are known to efficiently protect against this kind of damage [32]. In our study, we did not observe significant changes in the activity of transaminase in animals fed diets with beetroot crisps in comparison to the control groups. Mekkawy [33] observed its increase in a 30-day experiment with rats fed synthetic and natural dyes, as well as beet red-supplemented diet (0.08 and 0.4 g/kg diet). From the present results, we did not observe a higher antioxidant activity of diets containing beetroot crisps; neither the activity of SOD and GPx enzymes nor the total antioxidant status was significantly changed compared to the control group. Nevertheless, many *in vitro *studies with betalains from red beets have demonstrated that they possess high antiradical and antioxidant activity [5,7,13,34]. Lu and co-authors [35] found that the oral administration of betalains from red beets (5, 20 or 80 mg/kg BW) in irradiated mice significantly enhanced the activity of SOD and GPx in the liver, spleen and kidney in a dose-dependent manner. In three different experimental tumour models in mice, Kapadia [36] reported that a very low dose of betanin (0.0025%) from beetroot acts as a potent chemopreventive agent.

From the present study, it becomes clear that feeding of the 3% beetroot crisps-supplemented diet decreased hepatic total cholesterol of rats. The dyslipidaemic diet elicited a substantial accumulation of total cholesterol level in the liver and increased hepatic triglycerides concentration. In the experiment Moundras [24] a similar tendency has been reported.

## Conclusions

In conclusion, our current study demonstrates that beetroot crisp is a healthy food due to the diminution on total serum cholesterol and triacylglycerols levels in rats fed dyslipidaemic diets. Beetroot crisps seems clearly to modify caecum activity in rats fed experimental diets, altering intensification of the ceacum fermentation of rats and have a tendency to reduce blood glucose. It appears that the beetroot crisps should be consumed more often because of its health-promoting properties.

## Abbreviations

(SCFA): short chain fatty acids; (AST): aspartate aminotransferase; (HDL): high density lipoprotein cholesterol; (TC): total cholesterol; (ALT): alanine aminotransferase; (BW): body weight; (SOD): superoxide dismutase; (GPx): glutathione peroxidase; (TAS): total antioxidant status; (ABTS): 2,2'-azino-bis(3-ethylbenzthiazoline-6-sulphonic acid); (S): standard groups; (D): dyslipidemic groups; (B_0_): groups without beetroot crisps; (B_0.3_), (B_1_), (B_3_): groups with 0.3, 1 and 3% beetroot crisps respectively.

## Competing interests

The authors declare that they have no competing interests.

## Authors' contributions

MW carried out the experimental work, data collection and evaluation; performed the statistical analysis, literature search and draft preparation; and refined the manuscript for publication. JJ supervised the work and was responsible for critical review, intellectual input in the discussion and overall presentation of the paper. WW carried out an examination of betalains. All authors have read and approved the final manuscript.
